# HIV Incidence and Transactional Sex Among Men Who Have Sex With Men in Ningbo, China: Prospective Cohort Study Using a WeChat-Based Platform

**DOI:** 10.2196/52366

**Published:** 2024-07-23

**Authors:** Hang Hong, Xiaojun Shi, Yuhui Liu, Wei Feng, Ting Fang, Chunlan Tang, Guozhang Xu

**Affiliations:** 1 School of Public Health, Health Science Center, Ningbo University, Ningbo, China; 2 Beilun Center for Disease Control and Prevention, Ningbo, China; 3 Ningbo Center for Disease Control and Prevention, Ningbo, China; 4 Fenghua Center for Disease Control and Prevention, Ningbo, China

**Keywords:** HIV/AIDS, incidence, men who have sex with men, MSM, transactional sex, WeChat, HIV, STI, STD, sexual, behavior, behavioral, risk, risky, risks, China, Chinese, testing, mHealth, mobile health, app, apps, applications, applications, text message, text messages, messaging, social media, regression, sexually transmitted infection, sexually transmitted disease

## Abstract

**Background:**

Sexual transmission among men who have sex with men (MSM) has become the major HIV transmission route. However, limited research has been conducted to investigate the association between transactional sex (TS) and HIV incidence in China.

**Objective:**

This study aims to investigate HIV incidence and distinguish sociodemographic and sexual behavioral risk factors associated with HIV incidence among MSM who engage in TS (MSM-TS) in China.

**Methods:**

We conducted a prospective cohort study using a WeChat-based platform to evaluate HIV incidence among Chinese MSM, including MSM-TS in Ningbo, recruited from July 2019 until June 2022. At each visit, participants completed a questionnaire and scheduled an appointment for HIV counseling and testing on the WeChat-based platform before undergoing offline HIV tests. HIV incidence density was calculated as the number of HIV seroconversions divided by person-years (PYs) of follow-up, and univariate and multivariate Cox proportional hazards regression was conducted to identify factors associated with HIV incidence.

**Results:**

A total of 932 participants contributed 630.9 PYs of follow-up, and 25 HIV seroconversions were observed during the study period, resulting in an estimated HIV incidence of 4.0 (95% CI 2.7-5.8) per 100 PYs. The HIV incidence among MSM-TS was 18.4 (95% CI 8.7-34.7) per 100 PYs, which was significantly higher than the incidence of 3.2 (95% CI 2.1-5.0) per 100 PYs among MSM who do not engage in TS. After adjusting for sociodemographic characteristics, factors associated with HIV acquisition were MSM-TS (adjusted hazard ratio [aHR] 3.93, 95% CI 1.29-11.93), having unprotected sex with men (aHR 10.35, 95% CI 2.25-47.69), and having multiple male sex partners (aHR 3.43, 95% CI 1.22-9.64) in the past 6 months.

**Conclusions:**

This study found a high incidence of HIV among MSM-TS in Ningbo, China. The risk factors associated with HIV incidence include TS, having unprotected sex with men, and having multiple male sex partners. These findings emphasize the need for developing targeted interventions and providing comprehensive medical care, HIV testing, and preexposure prophylaxis for MSM, particularly those who engage in TS.

## Introduction

Treatment as a prevention strategy has been proven highly effective in reducing the risk of HIV transmission [1-3]. At the end of 2022, a total of 39.0 million people were living with HIV/AIDS globally, with 1.3 million new HIV infections, representing a 59% reduction since the peak in 1995 (3.2 million) and a 38% reduction relative to 2010 (2.1 million) [4]. However, men who have sex with men (MSM) bear a disproportionate burden of HIV infection compared with the general population [5]. The national surveillance system in China reported that sexual transmission among MSM had become the primary route of HIV transmission [6]. A large-scale systematic analysis found that MSM formed a high-risk population for HIV infection in China, with an overall national prevalence estimated to be 5.7% (95% CI 5.4%-6.1%) from 2001 to 2018. Furthermore, this study also identified an increasing trend in HIV prevalence over time [7]. As a potential bridge population for HIV transmission due to their potential sexual interactions with both high-risk clients and low-risk regular partners, the HIV epidemic among MSM is a significant public health issue in China [8].

Previous studies have demonstrated that factors associated with HIV infection among MSM include unprotected anal sexual behavior, meeting the last sexual partner, and testing positive for sexually transmitted diseases. However, most studies about HIV among MSM have primarily used cross-sectional designs [9-11]. Conducting prospective cohort studies is recommended to estimate HIV incidence, as they provide more precise and reliable data to monitor trends in the HIV epidemic and to evaluate the effectiveness of HIV prevention and control efforts [12,13]. An open cohort study in Beijing, the capital of China, observed an HIV incidence of 5.9 (95% CI 4.6-7.6) per 100 person-years (PYs) among MSM with an overall retention rate of 69.7% (699/1003) from 2009 to 2012 [14]. Unsurprisingly, loss to follow-up in some cohort studies was high due to the stigma and discrimination faced by MSM. A cohort study with 3512 MSM observed an HIV incidence of 3.6 per 100 PYs, with 2826 MSM completing the 68 monthly follow-ups in the city of Heilongjiang, China, during 2013‐2018. The retention rate of this study was low due to added pressure from families or wives of MSM who were married or cohabiting with female partners [15]. Therefore, it is necessary to develop new strategies to recruit and retain cohorts among MSM. WeChat has become one of China’s most widely used social media apps, with over 1.3 billion active users in 2022 [16], similar to a combination of Twitter, WhatsApp, and Facebook [17]. It shows promise as a platform for recruitment and has been used in HIV prevention studies [18,19]. However, there is limited research on using WeChat to maintain a cohort among MSM.

Transactional sex (TS) is broadly defined as the exchange of sex for money or goods [20]. Different studies have reported that MSM who engage in TS (MSM-TS) may be more likely to experience various forms of violence (emotional, physical, and sexual), use condoms inconsistently, engage in substance abuse, and experience psychological distress [21-23]. Worldwide, MSM-TS contributes to the HIV epidemic. A 2015 global meta-analysis showed an HIV prevalence among MSM-TS of 45.8% in South Africa, 19.3% in the United States, 27.3% in Peru, 14.7% in India, and 19% in Thailand [24]. A recent study conducted in China showed that MSM-TS were 2.7 times more likely to be infected with HIV compared with MSM who do not engage in TS (MSM-NTS) [25]. MSM-TS in China is a hidden and understudied population, with limited data on HIV infection and the underlying causes of engaging in TS among MSM from cohort studies.

This study aimed to investigate the HIV incidence among MSM-TS from 2019 to 2022 in Ningbo and to identify and distinguish sociodemographic and sexual behavioral risk factors associated with HIV incidence. We expect our findings to inform evidence-based HIV intervention among MSM-TS in China.

## Methods

### Study Design and Population

A prospective cohort study was conducted in Ningbo from July 2019 until June 2022. Participants were followed up approximately every 6 months after recruitment. The study end point was HIV seroconversion or June 2022, whichever came first. Eligibility criteria for study enrollment included (1) being at least 18 years old, (2) being a male who had oral or anal sex in the last year, (3) being HIV-negative at baseline, and (4) being willing to provide written informed consent to attend the follow-up survey.

### Recruitment Method

Baseline and follow-up visits were conducted through a combination of online and offline methods by the local Center for Disease Control (CDC) and community-based organizations. The recruitment process was as follows: (1) we developed a WeChat-based HIV counseling and testing appointment platform named Liu Se Yang Guang Fu Wu (LSYGFW); (2) the QR code of the platform was posted on Blued, Weibo, WeChat, websites, and MSM venues by local community-based organizations; (3) participants made appointments by searching for the nearest HIV testing site and determining the appointment time and place on LSYGFW; (4) participants signed the informed consent form on LSYGFW, which introduced the content of the investigation and emphasized the confidentiality and security of the HIV testing process; (5) after completing the self-administered questionnaire, participants received notification of the appointment completion and obtained the appointment number from LSYGFW; (6) trained investigators at the HIV testing site were responsible for checking appointment numbers, inclusion criteria, and the quality of the web-based questionnaire before HIV testing; (7) participants accessed LSYGFW to review test reports and receive health advice; (8) participants received LSYGFW messages containing a link to the follow-up questionnaire 6 months later and scheduled an appointment for the subsequent HIV tests; and (9) staff at the testing site guided those who came for testing without an appointment to make an on-site appointment.

### Questionnaire

The questionnaire included two parts: (1) sociodemographic characteristics, such as age, current location of residence (Ningbo or Non-Ningbo), duration of local residence, education, marital status, and monthly income, and (2) high-risk behaviors, such as sexual orientation, unprotected sex with men, multiple male sex partners (having 2 or more male sexual partners in the past 6 months), group sex with men (having participated in sexual activities with more than 2 men at the same time), sex with men after drinking alcohol, gay mobile app use, HIV testing, and synthetic drug use (having used any synthetic drugs such as methamphetamine, ketamine, and ecstasy).

### HIV Testing

HIV infection status was initially screened using a blood rapid detection reagent (Wan Fu Biotechnology Co, Ltd). For participants who tested positive, 5 mL of blood was collected and sent to the local CDC laboratory to test for HIV antibodies using an enzyme-linked immunosorbent assay (ELISA; Livzon Pharmaceutical Group Inc). Confirmation of HIV infection diagnosis was established using Western blot (HIV 2.2 WB; Genelabs Diagnostics).

### Statistical Analysis

EpiData software (version 3.1; The EpiData Association) was used for data storage, and R software (version 4.3.0; Bell Laboratories) was used for data analysis. Categorical variables were reported as numbers and percentages, while differences were tested using *χ*^*2*^ tests. Age data were presented using the median and IQR since they were not normally distributed. HIV incidence density and 95% CI were calculated as the number of HIV seroconversions divided by PYs over the observed time among those tested HIV-negative at baseline, assuming the exact Poisson method. This method is appropriate for rare events, such as HIV incidence, and accounts for the uncertainty in the estimated rates due to the limited number of events and person-time observed [26]. The cumulative incidence of HIV infection was estimated by Kaplan–Meier methods. We used univariate and multivariate Cox proportional hazards regression to identify factors associated with HIV incidence. *P* values of <.05 for 2-sided tests were considered statistically significant.

### Ethical Considerations

This study was reviewed and approved by the ethics board of the Ningbo CDC (201908). Informed consent was obtained from each participant. Participants were given a cash gift of ¥50 (approximately US $7) after each survey visit as compensation for their time. All participants found to be HIV-positive were referred to treatment.

## Results

### Enrollment

**Figure 1.**
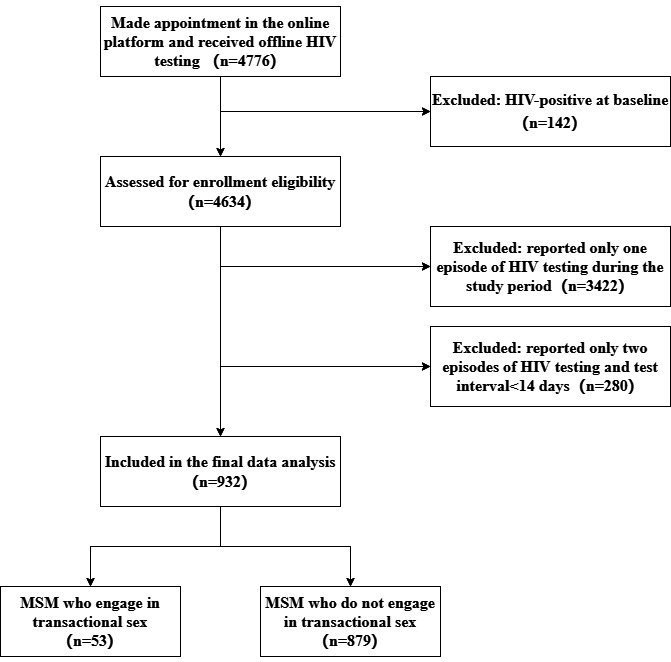
Flowchart of participants enrolled in the prospective cohort study from 2019 to 2022. MSM: men who have sex with men.

From July 2019 to June 2022, a total of 4776 participants made appointments on the web-based platform and received offline HIV testing, from which 142 participants were found to be HIV-positive at baseline and therefore excluded. Of the remaining 4634 HIV-negative MSM assessed for eligibility, 3422 were excluded for only having 1 HIV test during the study period, not meeting the follow-up requirement. An additional 280 were excluded for having only 2 HIV tests that were less than 14 days apart, which was considered an insufficient follow-up interval. After applying these eligibility and exclusion criteria, a total of 932 eligible participants were included in the final analysis. Figure 1 shows the detailed study selection process.

### Participant Characteristics

The median age of the 932 respondents was 27.0 (IQR 23.0‐33.0) years old. Most participants (733/932, 78.7%) were 18-34 years old, 57% (n=531) had registered local households, 58.9% (n=549) had lived in Ningbo for at least 2 years, 52.8% (n=492) had a college degree or above, 73.1% (n=681) were single, 52.7% (n=441) had an income below ¥5000 (US $700) per month, and 67.6% (n=542) self-identified as homosexual men (Table 1).

A total of 788 (84.5%) participants reported using gay apps, of which 604 (86.4%) reported having HIV testing at least once in their lifetimes, and 575 (82.3%) had been tested in the past 6 months. In total, 38.7% (n=361) of participants had unprotected sex with men, 41% (n=382) had multiple male sexual partners, 8.3% (n=77) had group sex with men, 14.3% (n=133) had sex with men after drinking alcohol, and 13% (n=121) had substance use in the past 6 months (Table 1).

**Table 1.** Comparisons of characteristics between men who have sex with men (MSM) who engage in transactional sex (MSM-TS) and MSM who do not engage in transactional sex (MSM-NTS) in Ningbo, China, 2019‐2022.

Characteristics		All participants, n (%)	Subgroup comparison		Chi-square (*df*)	*P* value
			MSM-TS (n=53), n (%)	MSM-NTS (n=879), n (%)		
**Age (years)**					7.944 (3)	.047
	≈18	314 (33.7)	22 (41.5)	292 (33.2)		
	≈25	419 (45)	16 (30.2)	403 (45.8)		
	≈35	140 (15)	8 (15.1)	132 (15)		
	≥45	59 (6.3)	7 (13.2)	52 (5.9)		
**Current location of residence**					1.437 (1)	.23
	Ningbo	531 (57)	26 (49.1)	505 (57.5)		
	Non-Ningbo	401 (43)	27 (50.9)	374 (42.5)		
**Duration of local residence (years)**					5.676 (2)	.06
	<1	272 (29.2)	23 (43.4)	249 (28.3)		
	≈1	111 (11.9)	6 (11.3)	105 (11.9)		
	≥2	549 (58.9)	24 (45.3)	525 (59.7)		
**Education level**					9.087 (2)	.01
	Middle school or less	128 (13.7)	14 (26.4)	114 (13)		
	High school	312 (33.5)	19 (35.8)	293 (33.3)		
	College or above	492 (52.8)	20 (37.7)	472 (53.7)		
**Marital status**					6.207 (2)	.045
	Single	681 (73.1)	33 (62.3)	648 (73.7)		
	Married	193 (20.7)	18 (34)	175 (19.9)		
	Divorce or widower	58 (6.2)	2 (3.8)	56 (6.4)		
**Monthly income (yuan)^a^**					8.190 (2)	.02
	<5000	491 (52.7)	38 (71.7)	453 (51.5)		
	≈5000	341 (36.6)	12 (22.6)	329 (37.4)		
	≈10,000	100 (10.7)	3 (5.7)	97 (11)		
**Sexual orientation**					6.154 (3)	.10
	Heterosexuality	36 (4.5)	3 (5.7)	33 (3.8)		
	Homosexuality	542 (67.6)	31 (58.5)	511 (58.1)		
	Bisexuality	224 (24)	17 (32.1)	207 (23.5)		
	Uncertain	130 (13.9)	2 (3.8)	128 (14.6)		
**Gay mobile app use**					0.502 (1)	.48
	No	144 (15.5)	10 (18.9)	134 (15.2)		
	Yes	788 (84.5)	43 (81.1)	745 (84.8)		
**HIV testing in the lifetime**					1.133 (1)	.29
	No	241 (25.9)	17 (32.1)	224 (25.5)		
	Yes	691 (74.1)	36 (67.9)	655 (74.5)		
**HIV testing^b^**					0.274 (1)	.60
	No	338 (36.3)	21 (39.6)	317 (36.1)		
	Yes	594 (63.7)	32 (60.4)	562 (63.9)		
**Unprotected sex with men^b^**					3.594 (1)	.06
	No	571 (61.3)	39 (73.6)	532 (60.5)		
	Yes	361 (38.7)	14 (26.4)	347 (39.5)		
**Multiple male sex partners^b^**					0.043 (1)	.84
	No	550 (59)	32 (60.4)	518 (58.9)		
	Yes	382 (41)	21 (39.6)	361 (41.1)		
**Group sex with men^b^**					3.461 (1)	.06
	No	855 (91.7)	45 (84.9)	810 (92.2)		
	Yes	77 (8.3)	8 (15.1)	69 (7.8)		
**Sex with men after drinking alcohol^b^**					4.833 (1)	.03
	No	799 (85.7)	40 (75.5)	759 (86.3)		
	Yes	133 (14.3)	13 (24.5)	120 (13.7)		
**Synthetic drug user^b^**					0.795 (1)	.37
	No	811 (87)	44 (83)	767 (87.3)		
	Yes	121 (13)	9 (17)	112 (12.7)		

a A currency exchange rate of Chinese yuan ￥1=US $0.14 is applicable.

b In the past 6 months.

### Comparisons of Characteristics Between MSM-TS and MSM-NTS

As presented in Table 1, 53 (5.7%) participants were identified as MSM-TS, and 879 (94.3%) participants were MSM-NTS. Compared with MSM-NTS, MSM-TS were much younger (*P*=.047), had less schooling (*P*=.01), were married (*P*=.045), had lower monthly income (*P*=.02), and more often reported having sex with men after drinking alcohol (*P*=.03).

### HIV Incidence Density

**Figure 2.**
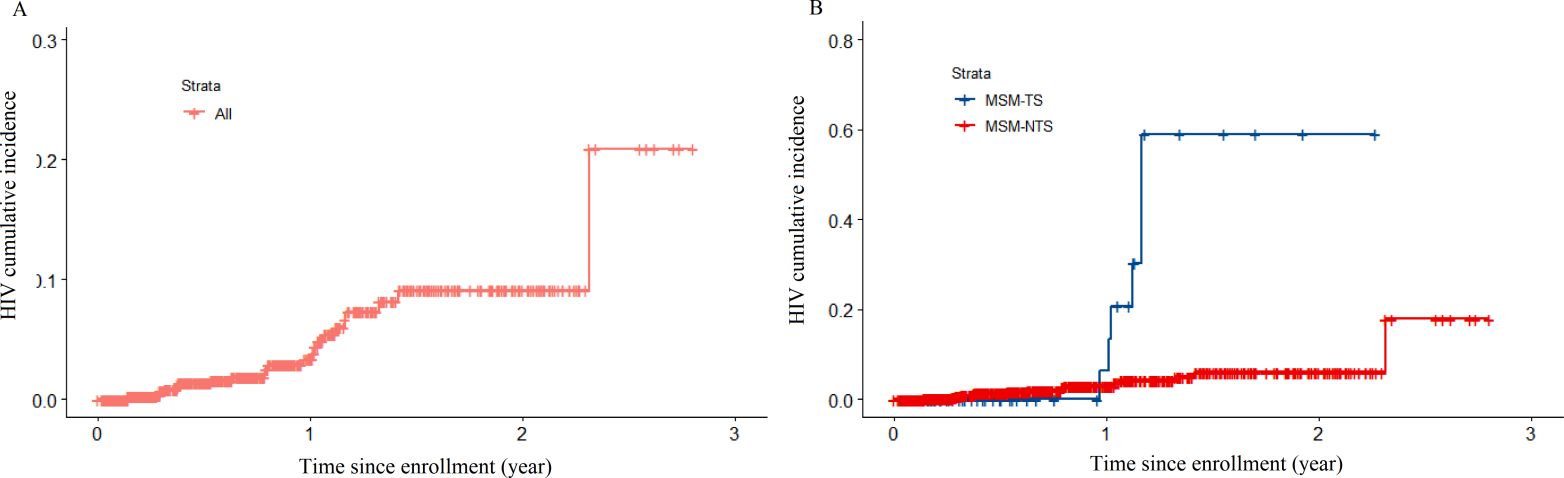
Kaplan–Meier cumulative incidence among MSM. (A) All MSM. (B) MSM-TS and MSM-NTS. MSM: men who have sex with men; MSM-NTS: MSM who do not engage in transactional sex; MSM-TS: MSM who engage in transactional sex.

During the study period, 25 HIV seroconversions were reported, with 630.9 PYs observed. The overall HIV incidence density was 4.0 (95% CI 2.7-5.8) per 100 PYs. The incidence of HIV among MSM-TS was 18.4 (95% CI 8.7-34.7) per 100 PYs, whereas it was 3.2 (95% CI 2.1-5.0) per 100 PYs among MSM-NTS (log-rank test=17.277; *P*<.001; Figure 2).

### Factors Associated With HIV Incidence

Univariate analysis showed that participants aged 25-34 years old (hazard ratio [HR] 0.29, 95% CI 0.11-0.82) had a lower HIV risk, with an incidence of 2.1 per 100 PYs, compared with those aged 18-24 years, with an incidence of 6.6 per 100 PYs. Those who engaged in unprotected sex with men in the past 6 months had a higher HIV incidence of 6.2 per 100 PYs (HR 7.63, 95% CI 1.80-32.42) compared with consistent condom users, with an incidence of 0.8 per 100 PYs. MSM-TS (HR 5.69, 95% CI 2.25-14.37) were more likely to acquire HIV than MSM-NTS (Table 2).

**Table 2.** Factors associated with HIV acquisition among men who have sex with men in Ningbo, China, 2019‐2022.

Characteristics		Number of follow-ups	Observed person-years	Number of seroconversions	Incidence per 100 person-years (95% CI)	Unadjusted HR^a^ (95% CI)	*P* value	Adjusted^b^ HR (95% CI)	*P* value
Overall		932	630.9	25	4.0 (2.7-5.8)	—^c^	—	—	—
**Age (years)**									
	≈18	314	183.1	12	6.6 (3.8-11.1)	1.00	—	1.00	—
	≈25	419	285.7	6	2.1 (1.0-4.5)	0.29 (0.11-0.80)	.02	0.20 (0.06-0.62)	.003
	≈35	140	111.6	4	3.6 (1.4-8.9)	0.51 (0.16-1.60)	.25	0.16 (0.03-0.82)	.03
	≥45	59	40.5	3	7.4 (2.6-19.7)	0.99 (0.28-3.57)	.99	0.19 (0.03-1.18)	.08
**Current location of residence**									
	Ningbo	531	362.5	10	2.8 (1.5-5.0)	1.00	—	1.00	—
	Non-Ningbo	401	258.4	15	5.8 (3.6-9.4)	2.22 (0.99-4.96)	.05	4.20 (1.30-13.59)	.02
**Duration of local residence (years)**									
	<1	272	137.3	5	3.6 (1.6-8.2)	1.00	—	1.00	—
	≈1	111	69.1	3	4.3 (1.5-12.0)	0.99 (0.23-4.15)	.98	1.63 (0.34-7.87)	.54
	≥2	549	414.5	17	4.1 (2.6-6.5)	0.93 (0.34-2.55)	.89	1.63 (0.27-9.69)	.18
**Education level**									
	Middle school or less	128	84.7	6	7.1 (3.3-14.6)	1.00	—	1.00	—
	High school	312	212.9	10	4.7 (2.6-8.4)	0.62 (0.22-1.70)	.35	0.59 (0.19-1.82)	.36
	College or above	492	323.3	9	2.8 (1.5-5.2)	0.37 (0.13-1.04)	.06	0.62 (0.18-2.07)	.43
**Marital status**									
	Single	681	449.7	16	3.6 (2.2-5.7)	1.00	—	1.00	—
	Married	193	131.0	8	6.1 (3.1-11.6)	1.76 (0.75-4.11)	.19	3.51 (0.82-15.05)	.09
	Divorce or widower	58	40.2	1	2.5 (0.4-12.8)	0.74 (0.10-5.60)	.77	1.79 (0.17-18.88)	.63
**Monthly income (yuan)^d^**									
	<5000	491	306.4	15	4.9 (3.0-7.9)	1.00	—	1.00	—
	≈5000	341	241.6	8	3.3 (1.7-6.4)	0.66 (0.28-1.56)	.35	1.06 (0.40-2.82)	.91
	≈10,000	100	72.9	2	2.7 (0.8-9.5)	0.56 (0.13-2.43)	.44	1.17 (0.23-6.07)	.85
**Sexual orientation**									
	Heterosexuality	36	23.1	1	4.3 (0.8-20.9)	1.00	—	1.00	—
	Homosexuality	542	384.8	18	4.7 (3.0-7.3)	0.97 (0.13-7.25)	.97	1.43 (0.15-13.81)	.76
	Bisexuality	224	136.2	4	2.9 (1.2-7.3)	0.65 (0.07-5.79)	.70	0.83 (0.07-9.54)	.88
	Uncertain	130	76.7	2	2.6 (0.7-9.0)	0.59 (0.05-6.54)	.67	0.65 (0.04-9.58)	.75
**Gay mobile app use**									
	No	144	96.3	3	3.1 (1.1-8.8)	1.00	—	1.00	—
	Yes	788	524.6	22	4.2 (2.8-6.3)	1.39 (0.41-4.66)	.60	1.07 (0.26-4.32)	.93
**HIV testing in the lifetime**									
	No	241	169.6	10	5.9 (3.2-10.5)	1.00	—	1.00	—
	Yes	691	451.3	15	3.3 (2.0-5.4)	0.61 (0.27-1.37)	.23	0.37 (0.04-3.62)	.39
**HIV testing^e^**									
	No	338	234.6	11	4.7 (2.6-8.2)	1.00	—	1.00	—
	Yes	594	386.3	14	3.6 (2.2-6.0)	0.85 (0.38-1.87)	.68	0.43 (0.05-3.76)	.44
**Transactional sex**									
	No	879	588.2	19	3.2 (2.1-5.0)	1.00	—	1.00	—
	Yes	53	32.7	6	18.4 (8.7-34.7)	5.69 (2.25-14.37)	<.001	3.93 (1.29-11.93)	.02
**Unprotected sex with men^e^**									
	No	361	246.8	2	0.8 (0.2-2.9)	1.00	—	1.00	—
	Yes	571	374.1	23	6.2 (4.1-9.1)	7.63 (1.80-32.42)	.006	10.35 (2.25-47.69)	.003
**Multiple male sex partners^e^**									
	No	550	367.3	13	3.5 (2.1-6.0)	1.00	—	1.00	—
	Yes	382	253.6	12	4.7 (2.7-8.1)	1.50 (0.68-3.30)	.31	3.43 (1.22-9.64)	.02
**Group sex with men^e^**									
	No	855	569.6	23	4.0 (2.7-6.0)	1.00	—	1.00	—
	Yes	77	51.3	2	3.9 (1.1-13.2)	0.86 (0.20-3.70)	.83	0.64 (0.12-3.32)	.60
**Sex with men after drinking alcohol^e^**									
	No	799	538.7	22	4.1 (2.7-6.1)	1.00	—	1.00	—
	Yes	133	82.2	3	3.7 (1.3-10.2)	0.93 (0.28-3.11)	.90	0.79 (0.20-3.15)	.74
**Synthetic drug user^e^**									
	No	811	539.8	24	4.5 (3.0-6.5)	1.00	—	1.00	—
	Yes	121	81.1	1	1.2 (0.2-6.7)	0.28 (0.04-2.11)	.22	0.16 (0.02-1.41)	.10

a HR: hazard ratio.

b Adjusted for age, current location of residence, duration of local residence, education level, marital status, monthly income, and sexual orientation.

c Not available.

d A currency exchange rate of Chinese yuan ￥1=US $0.14 is applicable.

e In the prior 6 months.

There was no multicollinearity in all variables, which had a collinearity tolerance greater than 0.1 or a variance inflation factor less than 5 (Table S1 in Multimedia Appendix 1). After adjusting for age, current location of residence, duration of local residence, education level, marital status, monthly income, and sexual orientation, factors associated with HIV acquisition were MSM-TS (adjusted HR [aHR] 3.93, 95% CI 1.29-11.93), having unprotected sex with men (aHR 10.35, 95% CI 2.25-47.69), and having multiple male sex partners (aHR 3.43, 95% CI 1.22-9.64) in the past 6 months. The sensitivity analysis adjusting only for age and education level showed that MSM-TS remained significantly associated with increased HIV incidence (aHR 2.48, 95% CI 1.19-5.18; Table 2).

## Discussion

### Principal Findings

HIV incidence refers to the number of new HIV infections in a population during a specific time frame and is a crucial indicator for HIV surveillance [27]. This study is a combination of an online and offline prospective cohort study, which aimed to evaluate HIV incidence and TS among Chinese MSM. We contribute to the existing research [14,20,25] by providing more accurate estimates of HIV incidence, particularly for MSM-TS. HIV incidence in this study among MSM-TS was significantly higher than among MSM-NTS. Our study found a correlation between being MSM-TS, engaging in unprotected sex with men, and having multiple male sexual partners in the past six months with higher HIV incidence.

In our study, MSM-TS tended to be younger and have less schooling than MSM-NTS. These results were consistent with another study in Latin America, which analyzed the link between HIV risky sexual behaviors and MSM-TS [28]. MSM-TS with less sexual experience and difficulty accessing information on safe sexual behaviors may be at increased HIV vulnerability based on age and educational level. Furthermore, our results showed that the monthly incomes of MSM-TS were much lower compared with another study among general MSM in the same city [29]. Using a social-ecological model to understand MSM motivations for TS and optimize related interventions is necessary. We found that MSM-TS were more likely to be married, indicating the potential bridges of HIV infection from MSM-TS to the general female population [30]. In addition, our results also showed that those consuming alcohol were significantly more likely to practice TS. This may be because MSM-TS undergo the burden of stigmatization of their sexual practices and the search for intense sexual sensations [31].

According to our study, the HIV incidence density stood at 4.0 per 100 PYs, which was consistent with the pooled HIV incidence rate (5.6 per 100 PYs) among Chinese MSM in a systematic review and meta-analysis conducted between 2015 and 2016 [32], 6.0 per 100 PYs in Chengdu from 2012 to 2018 [33], 5.3 per 100 PYs in sub-Saharan Africa from 2015 to 2016 [34], 4.7 per 100 PYs in Bangkok, Thailand from 2006 to 2010 [35], and 4.1 per 100 PYs in Chicago, United States between 2009 and 2015 [36]. However, HIV incidence among MSM-TS in this study was 18.4 per 100 PYs, demonstrating 5.7 times higher odds of HIV infection than MSM-NTS (3.2 per 100 PYs). This finding underscores the severe HIV burden faced by MSM-TS and the urgent need for targeted interventions and services for this population. A global meta-analysis estimated the pooled HIV prevalence among MSM-TS to be 19% in high-income countries and 24.5% in low- and middle-income countries, significantly higher than rates among MSM overall [24]. Another study conducted in China also found that MSM-TS had 2.7 times higher odds of HIV infection compared with MSM-NTS [7]. Luo et al [37] surveyed 259 MSM-TS in Hangzhou, China, and observed that the infection rate of HIV was 8.9%. Zheng et al [38] studied 252 MSM-TS and reported the prevalence of HIV to be 13.1% in six Chinese cities. Though these cross-sectional studies demonstrated that the prevalence of HIV was high among MSM-TS, only a few cohort studies have been conducted on this topic because MSM-TS are a marginalized and hard-to-reach population [39]. Our prospective cohort study also confirmed that TS could significantly increase the risk of HIV seroconversion in these MSM. The aHR associated with HIV seroconversion among MSM-TS was 3.93 when compared with MSM-NT, which was greater than the value of 3.38 estimated in Tianjin [25] or the value of 2.10 elevated risk in the survey conducted in Chongqing [40]. The global response to HIV/AIDS has largely overlooked the population of MSM-TS. In China, there are no national guidelines for monitoring and intervening in AIDS among MSM-TS, although interventions mainly target MSM and female sex workers. Compared with female sex workers, MSM-TS face unique challenges such as complex social networks, substance use, low condom usage, older sexual partners, and stigma [41]. New interventions should be developed to aid MSM-TS in delivering HIV prevention, testing, medical care, and preexposure prophylaxis (PrEP)/postexposure prophylaxis (PEP) to individuals with complex needs.

In addition to TS, we also found that having unprotected sex with men and multiple male sexual partners in the past 6 months was associated with a statistically elevated HIV incidence, as verified in many previous studies [12,32,42]. It suggested that effective HIV prevention and control strategies, such as distributing condoms and promoting peer education, may be necessary for managing transmission among the local MSM community. In addition, the use of gay mobile apps and its relation to the incidence of HIV have been topics of debate. Previous studies have reported that MSM who used apps had a higher frequency of male-male sexual contact, multiple male sexual partners, and unprotected sex with men [11]. However, some studies suggested that men who used apps were likelier to practice safe sex, were likelier to have better HIV testing habits, and were not at a higher risk for HIV [43]. In our study, using gay mobile apps had no causal association with HIV seroconversion. HIV incidence rates can vary due to ongoing interventions, changes in risk behaviors among certain groups, and differences in recruitment and follow-up methods [12].

To our knowledge, this was the first prospective cohort study using a WeChat-based platform to observe HIV infection and its risk factors, changes in risk behavior networks, and the relationship between web-based social interaction and HIV transmission in China. Additionally, WeChat-based questionnaires can enhance the quality of data gathered on sexual and stigmatized behaviors among MSM. Studies have shown that individuals tend to disclose more sensitive information when there is less personal interaction with the interviewer [44,45]. Therefore, there is great potential for using the WeChat-based platform in HIV research and intervention. The WeChat-based platform automatically enrolls new participants and allows follow-ups after HIV testing, leading to better participant retention and representation.

### Limitations

Our study is subject to several limitations. First, the limited number of MSM-TS in our study may affect the generalizability of findings for this subgroup. The convenience sampling methods used, while effective in engaging MSM, may underrepresent hidden populations like MSM-TS. This is a common challenge when studying marginalized or hard-to-reach populations, and future studies should consider alternative sampling strategies to ensure better representation of these groups. Second, the use of a WeChat-based platform for recruitment and follow-up in our study may have introduced some selection bias, as our cohort may overrepresent younger, more tech-savvy MSM compared with the broader MSM population. This limitation should be taken into account when extrapolating the findings to the general MSM community. Future research could explore the use of multiple recruitment methods to reach a more diverse sample of MSM. Lastly, the COVID-19 pandemic had a significant impact on our study, with many participants unable to attend scheduled HIV testing every 6 months due to restrictive measures. This resulted in follow-up interruptions, delays, and loss of follow-up, limiting our ability to assess the WeChat platform’s effectiveness in maintaining the MSM cohort. Despite these limitations, we believe it is important to report the pandemic’s impact on our study and discuss its potential effect on HIV incidence estimates. The disruptions caused by the pandemic highlight the need for flexible and adaptive research methods in the face of unforeseen circumstances. To mitigate the impact of similar disruptions in future research, innovative approaches should be considered. These may include the use of self-testing kits, web-based follow-up visits, or smartphone-based data collection. By incorporating these strategies, researchers can ensure the continuity of important HIV studies among MSM populations, even in the face of challenges such as those posed by the COVID-19 pandemic.

### Conclusions

This study revealed a high incidence of HIV among MSM-TS in Ningbo, China. The findings underscore the critical need for targeted interventions and comprehensive services tailored to the unique needs of this vulnerable subgroup. The identified risk factors associated with HIV incidence, including TS, having unprotected sex with men, and having multiple male sexual partners, highlight the importance of developing and implementing effective prevention strategies.

To address the disproportionate burden of HIV among MSM-TS, it is crucial to prioritize the development of innovative, culturally sensitive, and evidence-based interventions. These interventions should focus on increasing access to and uptake of essential services, such as regular HIV testing, PrEP, PEP, and comprehensive medical care. By providing these services in a nonjudgmental and inclusive manner, health care providers and public health professionals can work towards reducing HIV transmission and improving the overall health and well-being of MSM-TS.

## Supplementary material

10.2196/52366 Multimedia Appendix 1 Multicollinearity tests for all variables.

## Abbreviations

aHR: adjusted hazard ratio
CDC: Center for Disease Control
ELISA: enzyme-linked immunosorbent assay
HR: hazard ratio
LSYGFW: Liu Se Yang Guang Fu Wu
MSM: men who have sex with men
MSM-NTS: men who have sex with men who do not engage in transactional sex
MSM-TS: men who have sex with men who engage in transactional sex
PEP: postexposure prophylaxis
PrEP: preexposure prophylaxis
PY: person-year
TS: transactional sex
