# Supplementary material for: HIV Incidence and Transactional Sex Among Men Who Have Sex With Men in Ningbo, China: Prospective Cohort Study Using a WeChat-Based Platform
Source: JMIR Public Health Surveill. 2024 Jul 23;10:e52366. doi: 10.2196/52366 (PMC11287094; doi:10.2196/52366)
Supplement: Multimedia Appendix 1 [file publichealth-v10-e52366-s001.docx]

Table S1. Multicollinearity tests for all variables

| **Characteristics** | **Collinearity tolerance** | **Variance inflation factor** |
| --- | --- | --- |
|  |  |  |
| **Age(years)** | 0.698 | 1.433 |
| **Current location of residence** | 0.802 | 1.247 |
| **Duration of local residence(years)** | 0.847 | 1.180 |
| **Education level** | 0.757 | 1.321 |
| **Marital status** | 0.744 | 1.344 |
| **Monthly income(Yuan)** | 0.895 | 1.118 |
| **Sexual orientation** | 0.937 | 1.067 |
| **Gay mobile app use** | 0.954 | 1.048 |
| **HIV testing in the life time** | 0.348 | 2.869 |
| **HIV testing ^a^** | 0.370 | 2.706 |
| **Transactional sex** | 0.967 | 1.035 |
| **Unprotected sex with men ^a^** | 0.842 | 1.187 |
| **Multiple male sex partners ^a^** | 0.822 | 1.217 |
| **Group sex with men ^a^** | 0.835 | 1.198 |
| **Sex with men after drinking alcohol ^a^** | 0.922 | 1.085 |
| **Synthetic Drug User ^a^** | 0.882 | 1.134 |

^a^In the prior 6 months.
